# Overexpression of ATP Synthase Subunit Beta (Atp2) Confers Enhanced Blast Disease Resistance in Transgenic Rice

**DOI:** 10.3390/jof10010005

**Published:** 2023-12-21

**Authors:** Qiang Huang, Chunyan Chen, Xiyang Wu, Yingfei Qin, Xinqiu Tan, Deyong Zhang, Yong Liu, Wei Li, Yue Chen

**Affiliations:** 1 College of Plant Protection, Hunan Agricultural University, Changsha 410128, China; huangqiang131514@163.com (Q.H.);; 2 State Key Laboratory of Hybrid Rice, Institute of Plant Protection, Hunan Academy of Agricultural Sciences, Changsha 410125, Chinadyzhang78@163.com (D.Z.); haoasliu@163.com (Y.L.)

**Keywords:** transgenic, rice, ATP synthase subunit beta (Atp2), *Magnaporthe oryzae* (*M. oryzae*), reactive oxygen species (ROS)

## Abstract

Previous research has shown that the pathogenicity and appressorium development of *Magnaporthe oryzae* can be inhibited by the ATP synthase subunit beta (Atp2) present in the photosynthetic bacterium *Rhodopseudomonas palustris*. In the present study, transgenic plants overexpressing the *ATP2* gene were generated via genetic transformation in the Zhonghua11 (ZH11) genetic background. We compared the blast resistance and immune response of *ATP2*-overexpressing lines and wild-type plants. The expression of the Atp2 protein and the physiology, biochemistry, and growth traits of the mutant plants were also examined. The results showed that, compared with the wild-type plant ZH11, transgenic rice plants heterologously expressing *ATP2* had no significant defects in agronomic traits, but the disease lesions caused by the rice blast fungus were significantly reduced. When infected by the rice blast fungus, the transgenic rice plants exhibited stronger antioxidant enzyme activity and a greater ratio of chlorophyll a to chlorophyll b. Furthermore, the immune response was triggered stronger in transgenic rice, especially the increase in reactive oxygen species (ROS), was more strongly triggered in plants. In summary, the expression of *ATP2* as an antifungal protein in rice could improve the ability of rice to resist rice blast.

## 1. Introduction

The disease caused by the fungus *Magnaporthe oryzae* (*M. oryzae*), also known as rice blast, is the most destructive threat to rice production. Cultivation of disease-resistant rice varieties and chemical control are the main strategies utilized to prevent and control rice blast. However, inefficient management of fungal diseases using chemical fungicides can pose serious risks to the environment and food safety. To date, breeding and popularizing new disease-resistant varieties of rice are the most cost-effective, safe, and effective methods for controlling rice blast [1].

Although several resistance (*R*) genes from rice have been identified and cloned, only a few of them confer broad-spectrum and long-lasting resistance against rice blast disease and are frequently used in agriculture [2]. Since the *R* gene-conferring rice blast resistance does not cause genetic variation in the rice blast fungus [3], additional useful breeding approaches for the improving disease resistance and genetic resources are needed. In some cases, the heterologously expressed genes can enhance crop disease resistance, which is an effective strategy for disease resistance breeding. Numerous studies have demonstrated that introducing heterologous genes into rice can enhance rice blast resistance. For instance, immune-related genes, such as *RPW8.1* in *Arabidopsis* [4] and *Lr34* in wheat [5], can enhance transgenic rice resistance to *M. oryzae* through heterologous expression. Chitinase is a class of antifungal proteins that degrades chitin, which is the main component of fungal cell walls. To create new rice varieties resistant to the rice blast fungus, chitinase-coding genes from *Nicotiana tabacum* [6], *Momordica charantia* [7], and *Myxomycota* [8] have been transformed into rice, increasing rice disease resistance. Similarly, a number of other antifungal proteins, including ribosome inactivating protein (RIP) from *Momordica charantia* [9] and *Trichosanthes* [10], and oxalate decarboxylase protein from *Bacillus subtilis* [11], have been shown to be heterologously expressed in rice to enhance rice blast resistance. Interestingly, the silkworm moth *Cecropin A* gene, a key component of immune response in insects, has also been used to engineer resistance to rice blast fungus in transgenic rice [12]. Moreover, both the isoflavone synthase gene in soybean [13] and *CYP71Z18*, a gene that catalyzes maize phytoalexin biosynthesis [14], might affect phytoalexin metabolism to enhance the resistance to pathogens in transgenic rice lines. Therefore, utilizing antifungal active components from various biological sources is a useful approach to enhancing resistance to the rice blast fungus.

Throughout cellular life in living organisms, *ATP2* is essential for the transport of H^+^ by F1F0-ATPase, which is the catalytic site for F1F0-ATPase to catalyze the synthesis of adenosine 5′-triphosphate (ATP) [15]. In addition to ATP synthesis, *ATP2* is involved in other biological processes, such as lipid metabolism [16] tumor immune recognition [17], and influenza virus replication [18]. It is worth mentioning that the ATPase of plant cells is involved in the immune responses such as transmission of PAMP-triggered immunity (PTI) immune signals [19] and the production of reactive oxygen species [20]. However, the molecular mechanism through which Atp2 involved in plant-microbial interactions is currently unclear.

*Rhodopseudomonas palustris* (*R. palustris*) PSB06 is photosynthetic bacteria used as a biocontrol agent for plant disease [21]. The antifungal Atp2 protein, found recently in *R. palustris* PSB06 fermentation broth is the β subunit of F1F0-ATPase, and it limits the growth, appressorium formation and pathogenicity of *M. oryzae* [22]. In this study, to assess the effect of the Atp2 protein from *R. palustris* PSB06 on the resistance of rice to *M. oryzae*, *ATP2* was overexpressed in rice using *Agrobacterium tumefaciens*-mediated genetic transformation. The expression of *ATP2* in transgenic rice, reactive oxygen species (ROS) accumulation, and rice blast resistance were all investigated. The results suggest that *ATP2*, as an antifungal gene derived from biocontrol agents, can be utilized as an important genetic resource for controlling rice blast disease in the future.

## 2. Materials and Methods

### 2.1. Construction of the ATP2 Binary Vector for Transformation of Rice Plants

To generate *ATP2*-overexpressing plants, the *ATP2* CDS was cloned and inserted into the binary vector pU1301-Flag [23] under the 35S promoter and containing hygromycin phosphotransferase. In order to transform rice, the embryos of mature seeds of the rice variety Zhonghua11 (ZH11, Oryza sativa ssp. *japonica*) were used for callus induction, and the *ATP2* vector was subsequently transformed into rice calli using *Agrobacterium*. Non-*ATP2*-overexpressing plants was also transformed with the empty vector pU1301-Flag in the ZH11 genetic background The transformed callus tissues were selected on 50 mg/L hygromycin to obtain hygromycin-resistance plants. Then, we ascertained the positive plants by PCR and qRT-PCR. The PCR reaction parameters were 2 min at 95 °C, followed by 35 cycles of 30 s at 95 °C, 30 s at 56 °C, 2 min at 72 °C, and extension cycle of 5 min at 72 °C. The qRT-PCR reaction parameters were 94 °C for 2 min (one cycle), 94 °C for 30 s, and 60 °C for 3 s (40 cycles).

### 2.2. Evaluation of Blast Resistance

Fourteen-day-old seedlings were sprayed with the spore suspension (1.0 × 10^5^ spores/mL) of the *M. oryzae* strain Guy11. Photographs were taken 5–7 days after inoculation. Disease severity was evaluated by the lesion size on the inoculated leaves. The experiments were conducted with three biological replicates.

### 2.3. RNA Isolation and qRT-PCR Analysis

Total RNA was isolated from rice leaf tissue using the TRIzol reagent. After the analysis of the concentration and integrity of the total RNA, the RNA was subjected to cDNA synthesis using the cDNA Synthesis Kit (TransGen, Beijing, China). Then, the qPCR was carried out using a Green qPCR Kit (TransGen, Beijing, China) in an ABI 7500 Fast Real-Time PCR system (Applied Biosystems, Foster City, CA, USA).

The relative expression level of the genes was calculated based on CT values from the comparative 2^−ΔΔCt^ method. The rice *OsEF1α* gene, the reference gene, was used as a control for qPCR to analyze the expression of the *ATP2* gene and defense-related genes (*OsPBZ1*, *OsPAD4*, *OsLOX1*, *OsPR1a*, and *OsCHT1*). qRT-PCR was performed on three technical replicates. The qRT-PCR reaction parameters were 94 °C for 2 min (one cycle), 94 °C for 30 s, and 60 °C for 3 s (40 cycles).

### 2.4. Western Blot Analysis

Leaf tissues from two weeks old seedlings were homogenized in the extraction buffer containing: 2.5 mM EDTA, 250 mM Tris-HCl (pH 7.5), 0.1% ascorbic acid, and 1 mM PMSF. The extracted protein was then mixed with 5 × loading buffer containing 0.5% bromophenol blue, 10% SDS, 250 mM Tris-HCl, 50% glycerol, and 500 mM DTT. The mixture was boiled for five minutes, was separated on a 10% SDS-PAGE gel and electroblotted onto a nitrocellulose membrane, and subsequently probed with the anti-Flag antibody (AbM, Shanghai, China). The secondary antibody, goat anti-Mouse IgG HRP (AbM, Shsnghai, China), recognizes the primary antibody, which was detected with an HRP color development reagent.

### 2.5. ROS Burst Detection

Rice seedlings inoculated with *M*. *oryzae* were histochemically stained with nitroblue tetrazolium (NBT) and 3,3′-diaminobenzidine (DAB) to detect the in situ generation of hydrogen peroxide (H_2_O_2_) and superoxide radical (O^2−^), respectively [24]. The quantitative ROS levels in rice leaves inoculated with *M*. *oryzae* were also detected using the plant reactive oxygen species (ROS) ELISA kit (MSKBIO, Wuhan, China). The experiments were conducted in three biological replicates.

For the ROS burst in PTI immunity, the detection of ROS in rice leaf tissues induced by chitin based chemiluminescence. Here, 4 mm^2^ leaf disks of 4-week-old rice plants were incubated in sterile distilled water for 6 h. One reaction mixture contained 1 µL of 800 nM chitin, 1 μL of 1 μg/mL peroxidase-Streptavidin (HRP), 100 μL Immunstar-HRP substrate (Immun-star^TM^ HRP Luminol Enhancer, BioRad, Shanghai, China), and leaf disks. The reaction mixture for luminescence was measured in a Glomax 20/20 Luminometer (Promega, Beijing, China, Biotech). The experiments were conducted with three biological replicates.

### 2.6. Evaluation of Antioxidant Enzyme Activities

Leaf blades of the same weight from each sample were taken, ground in liquid nitrogen, and then homogenized with 10 mM PBS (pH 7.0). The obtained supernatant was collected as the crude enzyme extract from the rice leaves. The supernatant was collected by centrifugation at 12,000× *g* and 4 °C for 20 min and immediately used for the determination of superoxide dismutase (SOD), peroxidase (POD), and catalase (CAT) enzyme activities. The experiments were conducted with three biological replicates.

The SOD activity was determined by preventing the photochemical digestion of nitroblue tetrazolium (NBT) with a spectrophotometer and measuring the absorbance at 550 nm using the SOD activity assay kit (Solarbo, Beijing, China). The rate of absorbance changes at 550 nm when the absorbance is 50% inhibited in 1 mL of reaction buffer, which is the unit of SOD enzyme activity.

For POD, the reaction mixture contained 200 mL of PBS (pH 7.0), 0.076 mL of liquid guaiacol, and 0.112 mL of 30%H_2_O_2_. Fifty microliters of the enzyme extract and three milliliters of the reaction mixture were combined as one test reaction, and the solution’s absorbance of the solution at OD 470 nm was subsequently measured, with PBS used as the reference. The increase in absorbance of 0.01 at OD 470 nm per gram of fresh weight sample per minute is defined as one unit of POD enzyme activity.

The CAT activity was measured using the reaction mixture containing 200 mL of PBS (pH 7.0) and 0.3 mL of 1 nM H_2_O_2_. Fifty microliters of enzyme extract and three milliliters of the reaction mixture were combined, and the solution’s absorbance at OD 470 nm was subsequently measured, with PBS used as the reference. The activity of reducing 1 nM H_2_O_2_ per gram of fresh sample per minute at 240 nm was defined as one unit of enzyme activity.

### 2.7. Evaluation of Chlorophyll a and Chlorophyll b Contents

In liquid nitrogen, 0.1 g of leaf tissue was ground into powder and homogenized with 1 mL of 100% N, N-dimethylformamide (DMF), and centrifuged for 10 min; subsequently, the supernatant was collected, and the optical density was measured at 664 nm and 647 nm for chlorophyll a and chlorophyll b, respectively. The contents of chlorophyll a and chlorophyll b were calculated by the following formula: [chlorophyll a] = 12.7 × A_664_ − 2.79 × A_647_; [chlorophyll b] = 20.7 × A_647_ − 4.62 × A_664_. The experiments were conducted with three biological replicates.

### 2.8. Measurement of Growth Phenotype

The *ATP2*-overexpressing plants and their wild-type counterparts were grown in the greenhouse at 28 °C and 80% relative humidity under a light/dark cycle of 16 h/8 h. The seeds that had sprouted for three days were used to measure bud length; 14-day-old rice seedlings were used to measure root length and plant height; and the rice plants harvested in the field were dried at 40 °C for 3 days to measure the weight of 1000 grains. The experiments were conducted with three biological replicates.

## 3. Results

### 3.1. Genetic Transformation and Molecular Analysis of Transgenic Rice Plants Expressing ATP2

**Figure 1:**
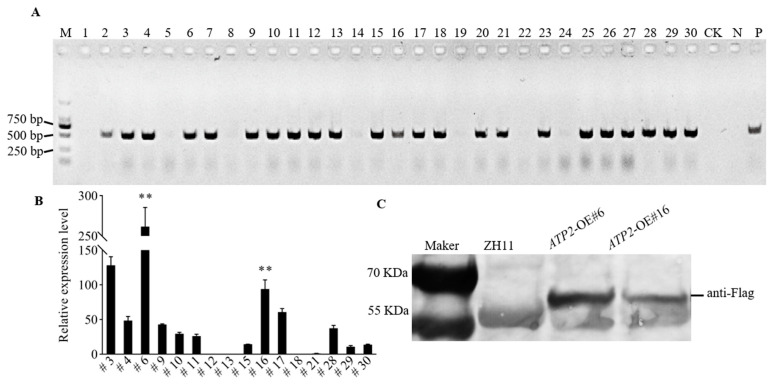
Expression level of *ATP2* in rice; (**A**) PCR analysis of plants transformed with *ATP2* using specific primers for the *hpt* gene; the DNA samples in lanes 1–30 were obtained from the 30 T1 transgenic plant lines used in the PCR analysis, representatively (M: DL2000 DNA markers, CK: the blank control, N: the negative control, and P: the positive control). (**B**) qRT-PCR analysis of *ATP2* expression in transformed plants relative to that of positive plant #21; (**C**) Expression levels of the Atp2 protein in wild-type ZH11 and two *ATP2*-overexpressing lines. Asterisks (**) show a significant difference between the wild-type plants used as controls and the transgenic plant (*p* < 0.01).

We first constructed an *ATP2* overexpression vector, which was subsequently used to generate 30 first-generation (T0) transformed plants through *Agrobacterium*-mediated genetic transformation. Twenty-three positive T1 plants were obtained after PCR screening with the hygromycin phosphotransferase gene (*hpt*) primer (Figure 1A), after which T2 generation seeds were bred and harvested from Hainan. The relative expression of *ATP2* was examined via qRT-PCR. Two T2-transformed plants (#6, #16.) selected for further study (Figure 1B) exhibited significantly higher (*p* < 0.01) expression levels relative to the positive plant #21, by 261-fold and 94-fold, respectively. Western blot analysis of two transgenic plants also showed that *ATP2* was effectively expressed in the transgenic rice plants (Figure 1C), which was verified by the detection of the FLAG-tagged protein.

### 3.2. Overexpression of ATP2 Could Confer Rice Blast Resistance in Transgenic Rice

**Figure 2:**
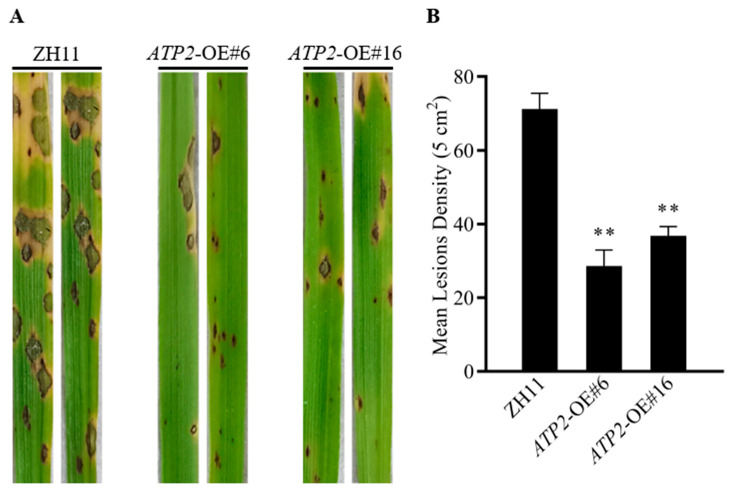
Effects of *ATP2* overexpression on rice blast resistance in rice seedling leaves; (**A**) Wild-type ZH11 and two *ATP2*-overexpressing lines; fourteen-day-old rice seedlings were sprayed with 1 × 10^5^/mL conidial suspension of the Guy11 strain. Inoculated rice seedling leaves were photographed at 7 dpi. (**B**) The lesion density was quantified by the number of lesions per unit leaf area. Asterisks (**) show a significant difference between the wild-type plants used as controls and the transgenic plants (*p* < 0.01). Three independent experiments were conducted.

The resistance of the transgenic rice plants to rice blast was assessed by spraying Guy11 onto two-week-old rice seedlings. At 7 days post-infection (dpi), as compared to those on wild-type plants, the lesion areas on *ATP2*-overexpression plants were noticeably reduced, particularly forming smaller and fewer lesions were observed on the transgenic rice plants (Figure 2A). The statistical results indicated that the lesion numbers of *ATP2*-OE#6 and *ATP2*-OE#16 was significantly lower (*p* < 0.01) by 43% and 35%, respectively (Figure 2B). The pathogenicity test of the plant transformed empty vector pU1301-Flag in the ZH11 genetic background was also conducted as a control against *ATP2*-overexpressing lines (#6, #16.). The results showed that the number of lesions on the positive plants was significantly lower (*p* < 0.01) than that in empty vector group (Figure S1). These results suggest that rice resistance to rice blast disease was directly enhanced by overexpressing *ATP2*, rather than indirect effects due to transgene expression.

### 3.3. Overexpression of ATP2 Has No Negative Impact on Rice Growth

**Figure 3:**
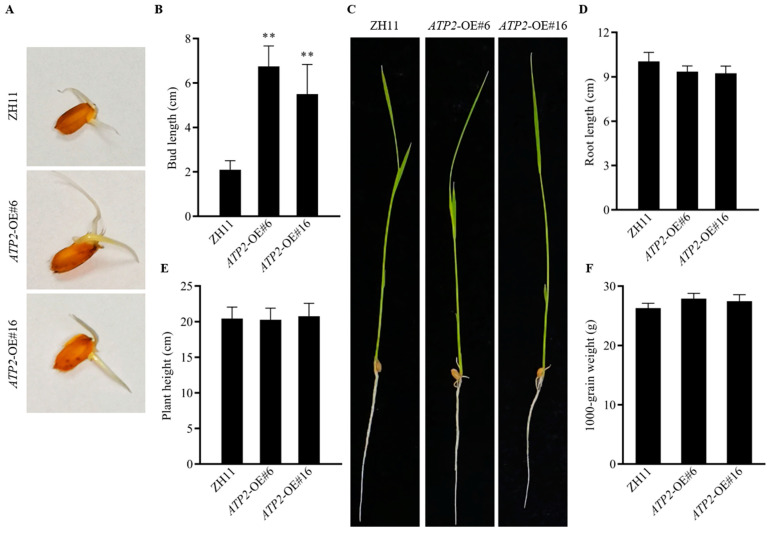
Phenotypes of the wild-type ZH11 and two *ATP2*-overexpressing lines; (**A**,**B**) The bud growth and bud length, respectively, of wild-type ZH11 and two *ATP2*-overexpressing lines at 3 days; (**C**–**E**) Root length, and plant height, respectively, of transgenic and wild-type plant seedlings at 14 days old; (**F**) 1000-grain weight of transgenic and wild-type plant in rice paddies; Asterisks (**) show a significant difference between the wild-type plants used as controls and the transgenic plants (*p* < 0.01). Three independent experiments were conducted.

The results of the phenotypic evaluation of the transgenic lines showed that the growth rate of the rice germination buds of wild-type plants was significantly lower (*p* < 0.01) than that of the transgenic lines. (Figure 3A,B). Furthermore, *ATP2* did not significantly influence the growth of roots (Figure 3C,D) or plant height (Figure 3C,E). Moreover, the thousand-gram weight of the rice plants (Figure 3F) harvested in the field also showed no significant difference (*p* < 0.01) in yield between the transgenic rice and wild-type plants. These results suggest that overexpression of the *ATP2* gene did not have a negative impact on the growth of rice.

### 3.4. Overexpression of ATP2 Strongly Promotes the Expression of Defense-Related Genes in Rice

**Figure 4:**
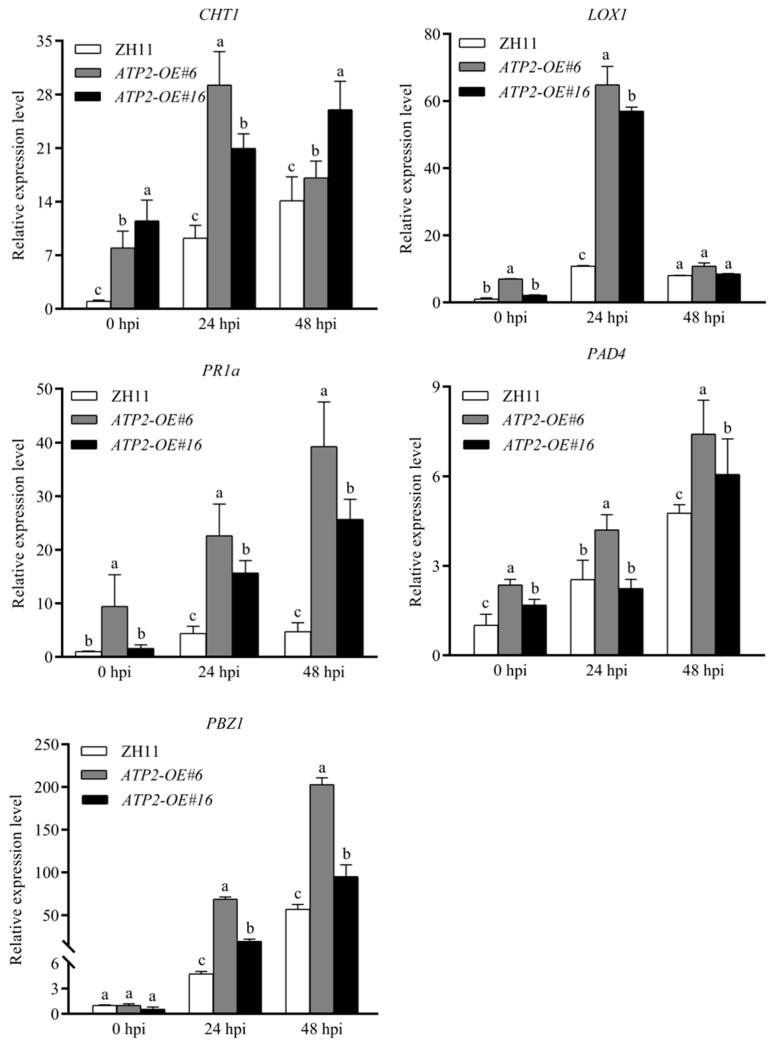
The expression levels of *PAD4*, *LOX1*, *PBZ1*, *PR1a*, and *CHT1* in the infected rice were assayed using qPCR. RNA samples were extracted from rice plants at 0 hpi, 24 hpi, and 48 hpi with the conidial suspension of *M. oryzae* strain Guy11. The average cycle threshold (Ct) values for triplicate reactions were normalized to the *EF1α* gene in rice. Significant differences can be seen when different letters are used for each treatment (*p* < 0.05). The data from three separate assays are provided as the mean ± SD.

After sensing the pathogen, plants activate multiple signal transduction pathways to initiate a series of complex defense reactions that require the participation of defense-related proteins. In this study, we examined whether plant defense-related genes were activated by analyzing the expression of the proteins *CHT1*, *PR1a*, and *PAD4* involved in the salicylic acid (SA) pathway and the genes *LOX1* and *PBZ1* regulated by the jasmonic acid (JA) pathway. After being infected with the rice blast fungus, the transgenic rice lines had significantly greater (*p* < 0.05) levels of transcription of defense-related genes than did the wild-type plants (Figure 4). Moreover, the expression of *LOX1* increased from 0 h to 24 h after inoculation but decreased from 24 h to 48 h, with the expression levels of two *ATP2* transgenic lines being significantly greater (*p* < 0.05) than those of the wild-type (Figure 4). These results showed that *ATP2*-overexpression significantly activated plant defense responses in rice.

### 3.5. Overexpression of ATP2 Affected Antioxidant Enzyme Activities and the Accumulation of Chlorophyll a and Chlorophyll b in Rice

**Figure 5:**
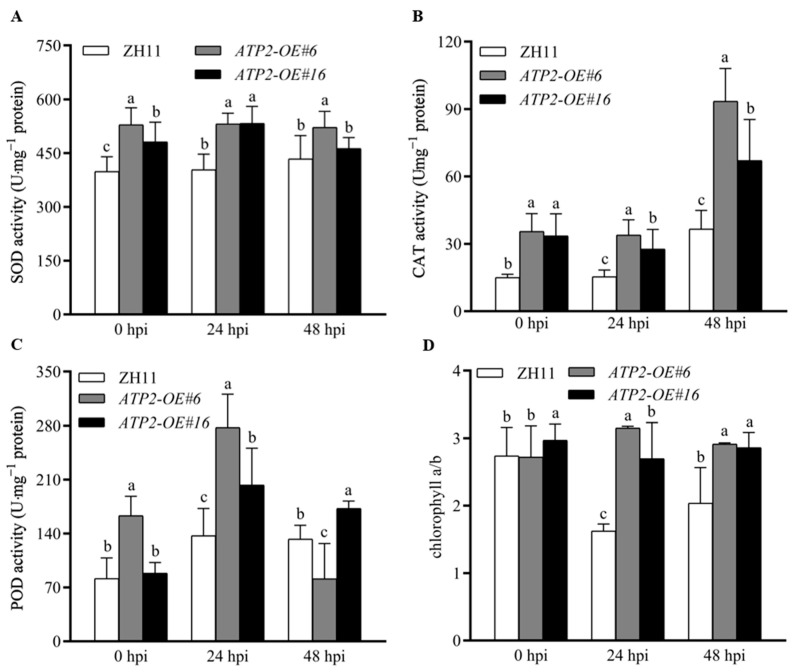
(**A**–**D**) Effects of *ATP2* overexpression on the activity of antioxidative enzymes, including SOD, CAT, POD, and chlorophyll a and b contents, respectively, in rice seedling leaves at 0 hpi, 24 hpi, and 48 hpi using a conidial suspension of the *M. oryzae* strain Guy11. Significant differences can be seen when different letters are used for each treatment (*p* < 0.05). The data from three separate studies are provided as the mean ± SD.

We measured the SOD, CAT, and POD enzyme activities and chlorophyll a and b contents in the leaves of the wild-type plants and *ATP2*-overexpressing lines. SOD, CAT, and POD enzyme activities in wild-type plants gradually increased as the infection time of the rice blast fungus changes from 0 hpi to 24 hpi and 48 hpi. Although the transgenic rice lines did not exhibit the same trend, their enzyme activity was significantly greater (*p* < 0.05) than that of the wild-type plants, whether they were infected with rice blast fungus or in a healthy state (Figure 5A–C). In the present study, both the transgenic and wild-type plants had a ratio of chlorophyll a to chlorophyll b close to 3, indicating that the plants were healthy. However, following the infection with *M. oryzae*, the ratio of chlorophyll a to chlorophyll b in the wild type decreased nearly twofold, while the *ATP2*-overexpression rice lines it was slightly changed during infection. (Figure 5D). These findings indicate that the expression of the Atp2 protein in rice enhances the activity of antioxidative enzymes and maintains the stability of chlorophyll synthesis.

### 3.6. Overexpression of ATP2 Affects the Accumulation of ROS in Rice

**Figure 6:**
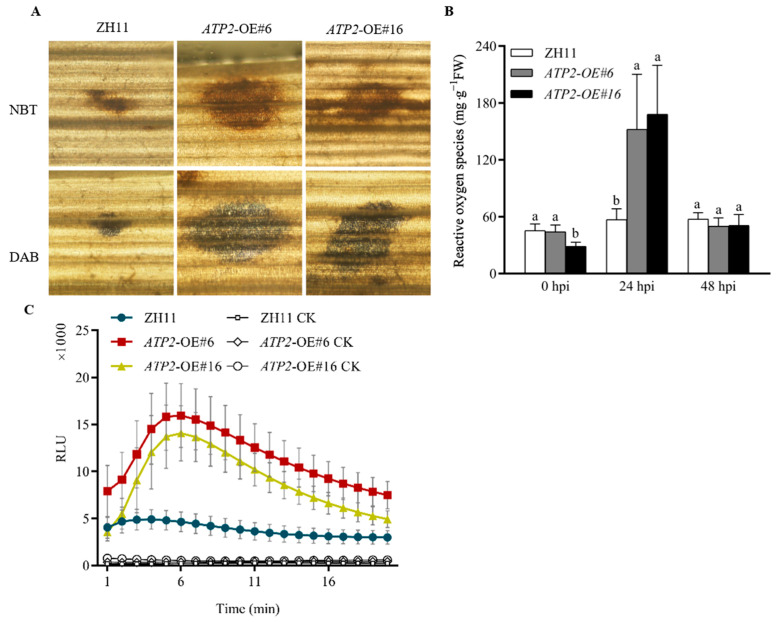
Effects of *ATP2* overexpression on the accumulation of ROS in rice leaves infected with the causal agent conidia of the *M. oryzae* strain Guy11; (**A**) NBT staining demonstrating the production and accumulation of O^2−^ in rice seedling leaves, and DAB staining demonstrating the production and accumulation of H_2_O_2_ in rice seedling leaves; (**B**) Quantitative determination of ROS accumulation in rice seedling leaves infected with conidial suspension of the *M. oryzae* strain Guy11, a causal agent of rice blast disease. Significant differences can be seen when different letters are used for each treatment (*p* < 0.05). The data from at least three separate studies are provided as the means ± SD. (**C**) Chitin (0.1 μg/mL) activates the ROS burst in rice leaves. H_2_O served as a negative control. The values are the means ± SD (*n* = 3). RLU, relative light units.

To regulate antioxidants and better respond to stress caused by the rice blast fungus, rice plants generate reactive oxygen species (ROS). The primary components of ROS, H_2_O_2_, and O^2−^ were examined to determine whether Atp2 has an impact on the accumulation of these compounds in plants and whether it impacts plant disease resistance. Although NBT staining caused the formation of blue formazan precipitates, indicating the accumulation of superoxide radicals, the appearance of dark brown precipitates as a result of DAB staining indicated the presence of hydrogen peroxide in the tissue. H_2_O_2_ and O^2−^ levels were significantly greater at the infection site in the leaves of *ATP2*-overexpressing rice plants than in those of wild-type plants at 24 hpi with conidial suspension (Figure 6A). ROS quantification results showed that the ROS burst in *ATP2* transgenic lines was significantly greater (*p* < 0.05) than that in the wild-type plants at 24 hpi (Figure 6B). In the process of the rice immune response to rice blast infection, chitin in the cell wall of rice blast fungus is recognized as a pathogen-associated molecular pattern (PAMP) that activates the PAMP-triggered immunity (PTI) in rice [25]. Therefore, we further tested the occurrence of ROS bursts in *ATP2* transgenic rice and wild-type plants under chitin-inducing conditions. Similarly, the burst of ROS activated by chitin treatment was significantly greater in *ATP2* transgenic rice than in wild-type plants, indicating that Atp2 enhanced the PTI activated by the rice blast fungus PAMP (Figure 6C). These findings suggest that the accumulation of ROS was greater in *ATP2* transgenic rice infected with the rice blast fungus than in control rice, which may fundamentally increase the resistance of the transgenic plants.

## 4. Discussion

In this study, heterologous expression of *ATP2*, instead of empty vector pU1301-Flag, significantly reduced the pathogenicity of *M. oryzae* in transgenic rice leaves, demonstrating that overexpression of *ATP2* directly enhanced rice blast resistance in transgenic rice and could lead to the use of this new genetic resource. Our previous study indicated that Atp2 could considerably inhibit the appressorial formation when *M. oryzae* conidia were treated, which showed strong antifungal activity against *M. oryzae* [22]. Moreover, in the present study, the overexpression of *ATP2* may improve immune responses in plants by controlling physiological metabolism and fundamental cellular processes. The basic function of Atp2, as a beta subunit of ATPase, is to participate in ATP synthesis, which is achieved by controlling pH changes and the membrane potential through ion transport in cells [26]. This effect may be mediated through the activation of signal transduction mechanisms involved in defense responses and ROS generation in rice, thereby strengthening the immune system.

During plant pathogen contact, a variety of signaling processes lead to the activation of various pathogenesis-associated protein production and defensive responses. In the present study, upon the infection of rice with *M. oryzae*, the stronger activation of JA- and SA-mediated defense responses caused by the overexpression of the *ATP2* gene was a significant cause of the inhibition of lesions in the transgenic rice plants, as indicated by the upregulated expression of defense-related genes. These defense response genes are expressed on the front line of defense against the pathogen. Chitinase is expressed by *CHT1* to hydrolyze chitin, the main component of pathogen cell walls [27]; *PAD4* is needed for the accumulation of terpenoid phytoalexins in rice [28]; while *LOX1* is necessary for the production of lipoxygenases in plants [29]; *PR1a* is expressed in correlation with systemic acquired resistance caused by pathogens in hosts [30]; and *PBZ1* is induced by the chemical, probenazole, induced rice disease resistance [31]. The severe limitation of the formation of disease lesions on the leaves of transgenic rice may be caused in part by immune system enhancement based on the significantly increased (*p* > 0.05) upregulated expression of these genes.

In addition to serving as a signal transducer to trigger other immune responses to fight pathogens [32], ROS can also damage infection hyphae within cells, halting the growth of *M. oryzae* [33]. Additionally, ROS perform crucial functions in plant growth, intercellular signaling and physiological and biochemical processes [34], and in coping with biotic and abiotic stresses. However, excessive generation of ROS in the cell can harm the plants [35], thus the primary antioxidant enzymes CAT, SOD, and POD play a critical roles in maintaining the balance of ROS generation [36]. In the present study, compared with those in wild-type plants, the ROS levels in the transgenic plants increased significantly (*p* > 0.05) after *M. oryzae* infection, as did the activity of antioxidative enzymes to maintain the internal ROS balance in the host at a moderate level. We further concluded that Atp2 enhanced PTI in transgenic plants when the ROS burst in transgenic plants increased significantly, induced by PAMP chitin in *M. oryzae*, which is the earliest immune response the host plant initiates [37].

As a beta subunit of ATPase, Atp2 participates in the synthesis of ATP, which occurs place in mitochondria and plasma membranes [38] and thereby produces ROS [39]. NADPH, which is involved in the production of ROS, is also needed for the function of ATPase activity [40]. Therefore, overexpression of *ATP2* from *R. palustris* may have affected the signaling and synthesis of ROS in rice infected with *M. oryzae*, resulting in an increased ROS burst.

According to various related studies, one of the important indicators of stress tolerance in rice is chlorophyll a and chlorophyll b [41], and infection by the rice blast fungus can lower the ratio of chlorophyll a to chlorophyll b content in the grain [42]. In the present study, Atp2 maintained the stability of chlorophyll synthesis in the transgenic plants, possibly improving the resistance of the rice plants to the rice blast fungus.

The longer bud length of the transgenic rice seeds was likely due to the overexpression of *ATP2*, which enhances the basic physiological and biochemical activities of the rice plants. Fundamental plant physiology and biochemistry are supported by the plant’s own ATPases, which are also recognized to be important in plant abiotic stress responses and immunity [43,44]. Therefore, the overexpression of *ATP2* may also serve as an additional contributor to the function of the plant’s own ATPase, improving the rice blast resistance to *M. oryzae*. However, it is still unclear how Atp2 is specifically involved mediating of the related crucial pathways in rice and whether bacterial Atp2 operates through the same mechanisms in plants. The present study suggested that the overexpression of the *ATP2* gene successfully elicited immune responses in rice and exerted no adverse effects on rice growth, further demonstrating its potential for application as a biocontrol agent against *M. oryzae*. Atp2, an antifungal protein isolated from biocontrol bacterial strains, can be used as an important genetic resource for the transgenic improvement of rice blast disease resistance in transgenic plants.

## 5. Conclusions

In the present work, we investigated resistance against *M. oryzae* in transgenic plants generated from novel rice cultivar using the antifungal protein Atp2. After transferring the *ATP2* gene was transferred from *R. palustris* PSB06 to the ZH11 rice cultivar, the expression levels of defense-related genes and the concentrations of immune-related metabolites, including antioxidant enzymes, chlorophylls, and ROS bursts induced by *M. oryzae* infection and chitin treatment significantly increased in transgenic rice, and rice development was not significantly negatively affected. In conclusion, the transgenic rice plants excelled from the wild-type rice plants in terms of blast disease resistance.

## Data Availability

Data are contained within the article and supplementary materials.

## Supplementary Materials

The following supporting information can be downloaded at: https://www.mdpi.com/article/10.3390/jof10010005/s1. Figure S1: Effects of *ATP2*-overexpression on rice blast resistance in rice seedling leaves; (A) Non-*ATP2*-expressing line (vector pU1301-Flag transform to ZH11) and two *ATP2*-overexpressing lines; fourteen-day-old rice seedlings were sprayed with 1 × 10^5^/mL conidial suspension of the Guy11 strain. Inoculated rice seedling leaves were photographed at 7 dpi. (B) The lesion density was quantified by the number of lesions per unit leaf area. Asterisks (**) show a significant difference between the wild-type plants used as controls and the transgenic plant (*p* < 0.05). Three independent experiments were conducted. Table S1: Primers used in this study.
