# Supplementary material for: Overexpression of ATP Synthase Subunit Beta (Atp2) Confers Enhanced Blast Disease Resistance in Transgenic Rice
Source: J Fungi (Basel). 2023 Dec 21;10(1):5. doi: 10.3390/jof10010005 (PMC10820023; doi:10.3390/jof10010005)
Supplement: Supplementary file 1 [file jof-10-00005-s001.zip › jof-2730467-supplementary.pdf]

Table S1. Primers used in this study.

primer	Primer Sequences (5'-3')	
21OV142-347R	TGATGGATGCCGCGAGT	Atp2 overexpression
21OV142-R	ATCATGATCTTTGTAATCGGATCCGGCCGCTTCGGCGG	vector construction
21OV142-F	CATTTACGAACGATAGCCGGTACCATGGCTACACCCGCCAACC	
Ubi-seqF	TAGCCCTGCCTTCATACGCT	
*hpt*557-F	ACACTACATGGCGTGATTTCAT	PCR and qRT-PCR
*hpt*557-R	TCCACTATCGGCGAGTACTTCT	Primer of rice
*Atp2*qpcr-F	GAGCGTACCCGTGAAGGTAA	transformation
*Atp2*qpcr-R	TTGTCCACGAAGAACAGCAC	
EF1α-F	CTTCAACACCCCTGCTATG	qRT-PCR Primer of
EF1α-R	CCGTTGTGGTGAATGAGTAA	Rice *EF1α*
LOX1-F	GCATCCCCAACAGCACATC	qRT-PCR Primer of
LOX1-R	AATAAAGATTTGGGAGTGACATATTGG	Rice LOX1
PAD4-F	GCCAGCTCCCCTACGACTTC	qRT-PCR Primer of
PAD4-R	CGTGTGCGGTGTAGGTTGTT	Rice *PAD4*
PBZ1-F	CTACTATGGCATGCTCAAGAT	qRT-PCR Primer of
PBZ1-R	ATAGAAAGGCACATAAACACAA	Rice *PBZ1*
Cht1-F	CGTGGTGACCAACATCATCA	qRT-PCR Primer of
Cht1-R	GAGTTGAAAGGCCTCTGGTTGT	Rice *Cht1*

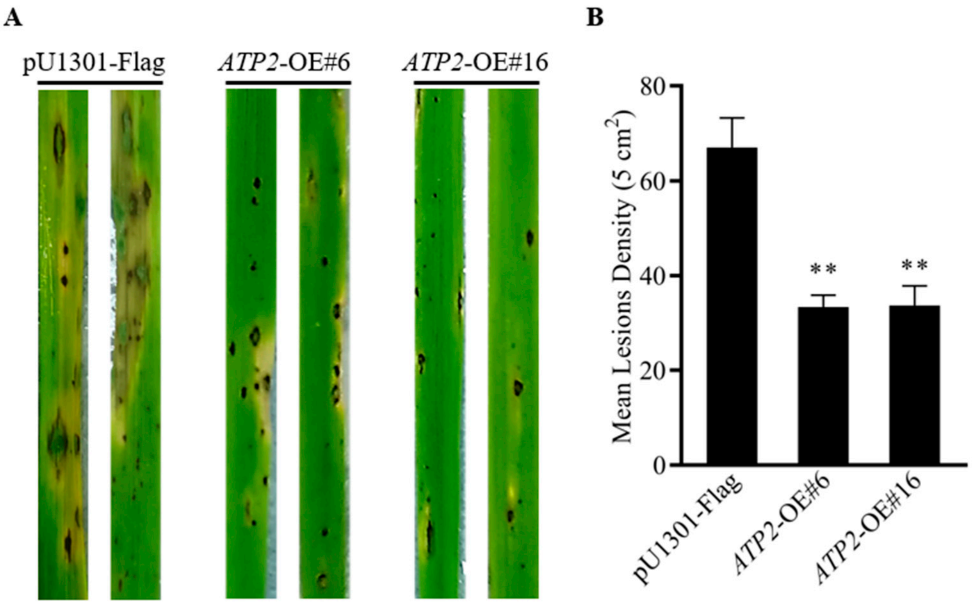

Figure S1. Effects of *ATP2* overexpression on rice blast resistance in rice seedling leaves; (**A**) Non-*ATP2*-expressing line (vector pU1301-Flag transform to ZH11) and two *ATP2*-overexpressing lines; fourteen-day-old rice seedlings were sprayed with 1×10^5/mL conidial suspension of the Guy11 strain. Inoculated rice seedling leaves were photographed at 7 dpi. (**B**) The lesion density was quantified by the number of lesions per unit leaf area. Asterisks (**) show a significant difference between the wild-type plants used as controls and the transgenic plant (p <0.05). Three independent experiments were conducted.
